# Extracellular vesicles at the crossroad between cancer progression and immunotherapy: focus on dendritic cells

**DOI:** 10.1186/s12967-024-05457-4

**Published:** 2024-07-29

**Authors:** Tiziana Schioppa, Carolina Gaudenzi, Giovanni Zucchi, Arianna Piserà, Yasmin Vahidi, Laura Tiberio, Silvano Sozzani, Annalisa Del Prete, Daniela Bosisio, Valentina Salvi

**Affiliations:** 1 Department of Molecular and Translational Medicine, University of Brescia, Viale Europa 11, Brescia, 25123 Italy; 2 IRCCS Humanitas Research Hospital, Milan, Italy; 3 Department of Molecular Medicine, Sapienza University of Rome, Laboratory Affiliated to Institute Pasteur- Italia, Rome, Italy

**Keywords:** Exosomes, Microvesicles, Antigen delivery, MHC, DC/NK crosstalk, Antigen presentation

## Abstract

Extracellular vesicles (EVs) are nanosized heat-stable vesicles released by virtually all cells in the body, including tumor cells and tumor-infiltrating dendritic cells (DCs). By carrying molecules from originating cells, EVs work as cell-to-cell communicators in both homeostasis and cancer but may also represent valuable therapeutic and diagnostic tools. This review focuses on the role of tumor-derived EVs (TEVs) in the modulation of DC functions and on the therapeutic potential of both tumor- and DC-derived EVs in the context of immunotherapy and DC-based vaccine design. TEVs were originally characterized for their capability to transfer tumor antigens to DCs but are currently regarded as mainly immunosuppressive because of the expression of DC-inhibiting molecules such as PD-L1, HLA-G, PGE2 and others. However, TEVs may still represent a privileged system to deliver antigenic material to DCs upon appropriate engineering to reduce their immunosuppressive cargo or increase immunogenicity. DC-derived EVs are more promising than tumor-derived EVs since they expose antigen-loaded MHC, costimulatory molecules and NK cell-activating ligands in the absence of an immunosuppressive cargo. Moreover, DC-derived EVs possess several advantages as compared to cell-based drugs such as a higher antigen/MHC concentration and ease of manipulation and a lower sensitivity to immunosuppressive microenvironments. Preclinical models showed that DC-derived EVs efficiently activate tumor-specific NK and T cell responses either directly or indirectly by transferring antigens to tumor-infiltrating DCs. By contrast, however, phase I and II trials showed a limited clinical efficacy of EV-based anticancer vaccines. We discuss that the future of EV-based therapy depends on our capability to overcome major challenges such as a still incomplete understanding of their biology and pharmacokinetic and the lack of standardized methods for high-throughput isolation and purification. Despite this, EVs remain in the limelight as candidates for cancer immunotherapy which may outmatch cell-based strategies in the fullness of their time.

## Background

All cells, including tumor and immune cells, release extracellular vesicles (EVs) delimited by double-leaflet lipid membranes which bear the potential to transfer bioactive molecules and molecular information to bystander cells, thus representing potent cell-to-cell communicators [1]. Indeed, EVs are the objects of a flourishing literature showing their capability to influence both homeostatic processes and diseases including cancer [2, 3]. In addition, EVs are regarded as promising therapeutic and diagnostic tools [4]. However, our knowledge on the biology of EVs remains limited and result generalization a major challenge [4–7].

Dendritic cells (DCs) are innate immune cells that master the inflammatory response by releasing both pro- and anti-inflammatory cytokines in response to danger signals, including pathogens and cancer cells, sensed by a vast array of innate receptors [8]. In addition, DCs also activate immune responses working as professional antigen-presenting cells (APCs) [9]: following pathogen sensing and DC activation, the antigen is up-taken, processed and presented to naïve T lymphocytes to prime antigen-specific immune responses. Depending on the pattern of costimulatory molecules expressed by DCs, this process may induce either tolerance or immunity [10].

Despite these common features, DCs comprise different subsets distinguished by ontogeny, phenotypical features, tissue distribution and transcriptional profiles (recently reviewed in [8, 11, 12]). Briefly, conventional or classical DCs (cDCs) include cDC1s and cDC2s. cDC1s specialize in cross-presentation of tumor-associated antigens to CD8^+^ T lymphocytes, thus shaping antitumor immune responses [13]. By contrast, cDC2s efficiently present antigens to CD4^+^ T cells and promote T helper cell polarization [14]. A single-cell analysis revealed further heterogeneity within cDC2, namely DC2 and DC3 [15, 16], which are undergoing further characterization [17, 18]. A second main subset are plasmacytoid DCs (pDCs), the major producers of type I interferons (IFNs) and masters in eliciting antiviral and antitumor immunity [19]. Finally, monocyte-derived DCs (moDCs) differentiate in response to inflammatory stimuli and are recruited to inflammatory sites, including the tumor micro-environment (TME) [20].

All DC subsets are central components of the TME, where they can promote antitumor adaptive immune responses but also tolerogenicity, where immunosuppressive factors alter their phenotype and effector functions [21–25]. Because of this dual role, DCs represent crucial decision makers in the response to immune checkpoint inhibitors as well as attractive targets for cancer immunotherapy [26, 27].

This review will focus on the role of tumor-derived EVs (TEVs) in the modulation of DC functions and on the therapeutic potential of both tumor- and DC-derived EVs. Given the complexity of EVs as biological entities and research/therapeutic tools, we also briefly summarize evidence to help critical interpretation of the existing findings and of their general significance. Consistent with this aim, particular attention was placed in selecting literature including sufficient detailing of experimental procedures of EV purification and characterization. When surveying the literature, “exosomes” is by far the most studied population of TEVs, but nomenclature is rather inconsistent, especially for works dating back several years. For this reason, the term EVs will be used throughout the review, with specifications of EV types whenever possible.

## Current knowledge of EV features

EVs are challenging to study because of their small size (mostly < 1 μm diameter), heterogeneity and substantial lack of discriminative markers [4]. Indeed, the biology, composition and even nomenclature of EVs are still debated and concerns remain on methods of purification, characterization and data reporting. This poses problems with generalization of published results, often discordant, as comprehensively reviewed elsewhere [4–7]. Here, we will only briefly summarize some major knowns and unknowns of EVs that need to be considered to critically review the available knowledge of how EVs influence DC functions in cancer and represent potential tools in cancer immunotherapy.

### EV types, size and origin

**Fig. 1:**
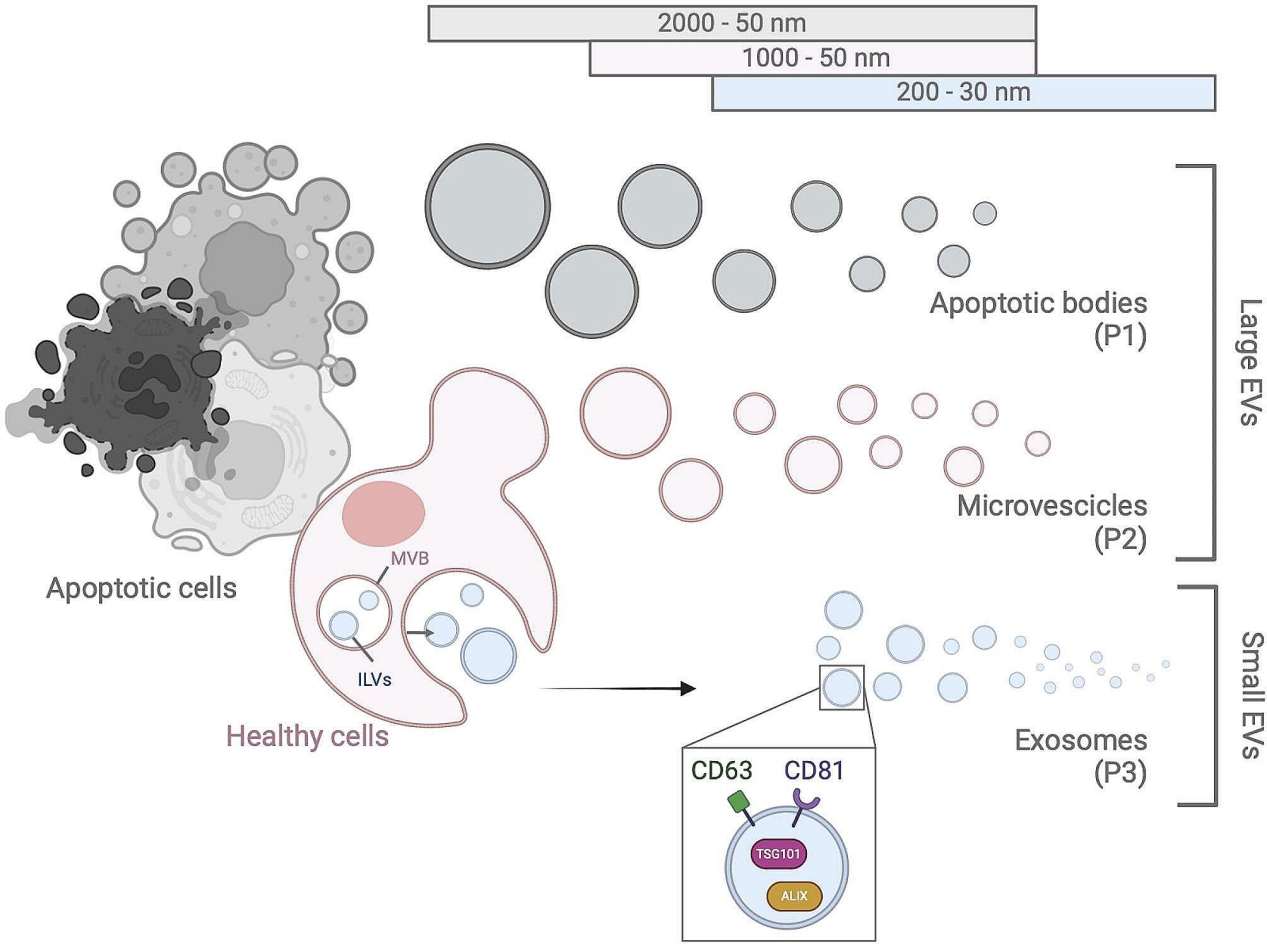
Biogenesis and classification of extracellular vesicle subtypes. The three different classes of EVs are depicted: apoptotic bodies are released through blebbing by cells undergoing apoptosis; microvesicles are generated by outward budding of the plasma membrane and exosomes are released by the fusion of multivesicular bodies (MVB) with the plasma membrane

The term EVs comprises a broad range of vesicles, that are usually classified based on their size and subcellular origin, but may also differ in terms of shape, density, membrane composition and surface molecules, internal cargo and originating cell type [5] (Fig. 1). Two major biogenetic sites identify two classes of EVs in living cells: exosomes (or ectosomes) and microvesicles (or microparticles) [28]. Exosomes originate as intraluminal vesicles (ILVs) upon inward budding of endosomal membranes, within the lumen of late endosomes, often indicated as multivesicular bodies (MVBs). Exosomes are then released by fusion of MVBs with the plasma membrane [29]. Microvesicles are instead generated via direct budding from the plasma membrane [29]. The same biogenetic pathway is shared by apoptotic bodies, a third type of EVs, with the difference that shedding occurs exclusively from apoptotic cells [30]. On average, depending on the biogenesis, EVs also differ in size: while exosomes display a mean diameter lower than 200 –150 nm, microvesicles and apoptotic bodies may reach or even overtake a diameter of 1000 nm. For this reason, the current nomenclature prefers the term “small EVs” for exosomes and “large EVs” for budding particles. However, relationships between size and origin of EVs are actually more complicated than this: for example, membrane budding EVs may display diameters less than 100 nm, thus representing “small microvesicles”, while heterogenous ILVs can give rise to a proportion of “large exosomes” [5]. Also, cell-type-specific differences in EV size were observed by some authors [31]. Thus, size alone cannot be used to definitively categorize EV subpopulations.

Theoretically, protein cargoes may shed light on the subcellular origin of EVs. For example, the presence of tetraspanins (e.g. CD63 and CD81) or components of the endosomal sorting complex (e.g. TSG101 and Alix) are used to identify endosome-derived EVs. However, these molecules also traffic through the plasma membrane and may decorate plasma membrane-derived EVs as well, although at lower levels [31]. Thus, these markers cannot precisely characterize the origin of EVs.

Currently, a combination of size and markers is used to categorize EVs although, as a matter of facts, we do not possess the technology to discriminate EV subtypes after they have been released in the extracellular space [32]. As a consequence of these difficulties, a lack of consensus between leading groups slowed down the publication of guidelines concerning EV nomenclature, allowing the usage of an eclectic mix of names for overlapping populations and of diverse names for the same population [33]. When surveying the literature, “exosomes” clearly result as the most studied EV population: this phenomenon seems to self-perpetuate leading to the assumption, not supported by current evidence, that they are more important and more interesting than other EV populations [34].

### EV cargo: how much and how delivered?

EVs potentially contain any cell component including membrane, cytosolic and nuclear proteins, metabolites, messenger RNAs (mRNAs) and several types of non-coding RNAs [4, 35, 36]. Of course, these components are not all present in each single EV: indeed, available results usually depict a population average content, while how different molecules are distributed in individual vesicles, or in vesicle subpopulations, is only beginning to being investigated by means of single-vesicle analysis techniques. Theoretically, the larger an EV, the more its content is represented by cytoplasmic entities, whereas smaller EVs are more likely to incorporate molecules located in close proximity to membranes. This concept implicates that cargo sorting mechanisms should prevail in small EVs unless extremely efficient packaging mechanisms exist [37]. However, since packaging mechanisms have not been described yet, it is reasonable to postulate that size constraint may apply. For example, large molecules such as mRNAs with associated proteins or big enzymatic complexes may not fit within small EVs. However, not all cargoes need to be contained in the EV lumen, as observed for nucleic acids associated with the outside of EV membranes [38], although doubts remain that this may represent an artifact correlated to specific isolation procedures [38].

EVs exert effects on target cells by several modalities, often combined together [1] including: (a) contact, such as that between surface proteins on EVs and on cells; (b) uptake, such as the case in which EVs are internalized in the endosomal compartment; and (c) fusion between EV- and cell-membranes, with release of the cargo into the cytoplasm. Because direct evidence of fusion is scarce, uptake is possibly the main mechanism of cargo delivery [39, 40]. Most questions concerning general mechanisms of EV uptake, however, remain unanswered, including how cargo is unloaded into the cell and in which compartment. The latter is particularly relevant when dealing with the delivery of biological molecules such as microRNAs or enzymes that need to remain correctly folded and active. Other uncertainties concern if different EV subpopulation undergo differential uptake pathways, if uptake depends on specific processing mechanism and its primary purpose such as cell communication, or mere disposal of byproduct of clearance, and many more.

When studying EV uptake, attention should be paid also to dose and time. Human serum small EVs were shown to be internalized by one specific or by several cell types depending on the dose [41], indicating that careful dose-dependent experiments should be planned in order to avoid misleading results. For what concerning time, EV uptake was shown to be very fast and consistent with endocytosis rates, requiring as little as 15 min [40]. As a consequence, experiments implying single administrations of EVs followed by long incubations may need to be rethought, unless dictated by the kinetic of the specific functional readout.

### Issues with EV preparation and reporting of EV-studies

Sequential ultracentrifugation, eventually followed by sucrose gradient, represents the gold standard for EV separation [5]. A detailed protocol was published in 2006 and further optimized in subsequent years [42]. In short, apoptotic bodies and cell debris are pelleted at 2,000 × g, microvesicles at 10,000 × g, while exosomes are pelleted at centrifugal forces of 100,000 × g and above (P1, P2 and P3 in Fig. 1, respectively). Notably, large differences in sedimentation patterns and efficiency were observed depending on the originating cell lines [43]. Even more relevant, yield and purity are greatly influenced by rotor type and centrifugation times [44]. Finally, ultracentrifugation may introduce artefacts such as aggregation, disruption of membrane topology and decoration of EVs with soluble components floating in the sample [45].

Other methods can be used to obtain EVs, all of them with different advantages and pitfalls [6, 7]. For example, filtration by gravity flow is gentler than ultracentrifugation: however, small-pored filter may trap EVs and pushing though the filter may damage or break membranes, which in turn may affect functionality [6]. Separation techniques may also perform differently depending on the biological sample or over-purify/select specific EV subpopulation. Moreover, the starting sample volume and type were suggested to influence EV stability and recovery [46].

There is also no universal agreement concerning the practices for EV counting and sizing [46]. Nanoparticle tracking analysis, tunable resistive pulse sensing, Raman spectroscopy, flow cytometry, single-particle interferometric reflectance, imaging sensing and electron microscopy are all used for counting and sizing, with different accuracy, resolution, strengths and weaknesses [7]. It is generally agreed that different measurement technologies are biased towards certain EV size ranges and that technical differences or protocol discrepancies among laboratories explain inadequate recovery of EVs with extremely small or large dimensions [5].

In this scenario, it is crucial to provide transparent reporting to allow for data replication, proper interpretation and finally reconciling conflicting data from different laboratories [47]. In addition to technical details concerning the methods of purification and characterization discussed above, there are many more aspects deserving careful reporting. For example, the passage number of cells and/or disease stage was shown to impact on the production of EVs in terms of number, cargo, and markers [48, 49]. Cell culturing in the presence or absence of bovine serum should be declared together with methods of EVs depletion from serum, given that complete removal of bovine contaminants is a tough duty [50]. On the other hand, cell culturing in serum-free conditions changes RNA profiles and promotes selection of clonal populations. Although Mycoplasma control is a very challenging issue, accurate Mycoplasma detection/removal in cell culture is mandatory because free Mycoplasma co-sediment with small EVs and may activate Pattern Recognition Receptors (PRRs) in sensitive cells [51]. Parameters such as kinetic or EV amount used for stimulation are rarely described, especially in in vitro systems, and if described may be difficult to interpret and/or to reproduce because of the lack of protocol standardization. All these aspects have obvious consequences on experimental outcomes and may make it difficult to draw conclusions as to at what extent the observed effects are due to experimental manipulation. Experimental consistency and detailed reporting are key to identify large-scale, significant results in the field of TEV biology and function.

## Tumor-derived EVs deliver antigens to DCs, but also impair their antigen-presenting capabilities

**Fig. 2:**
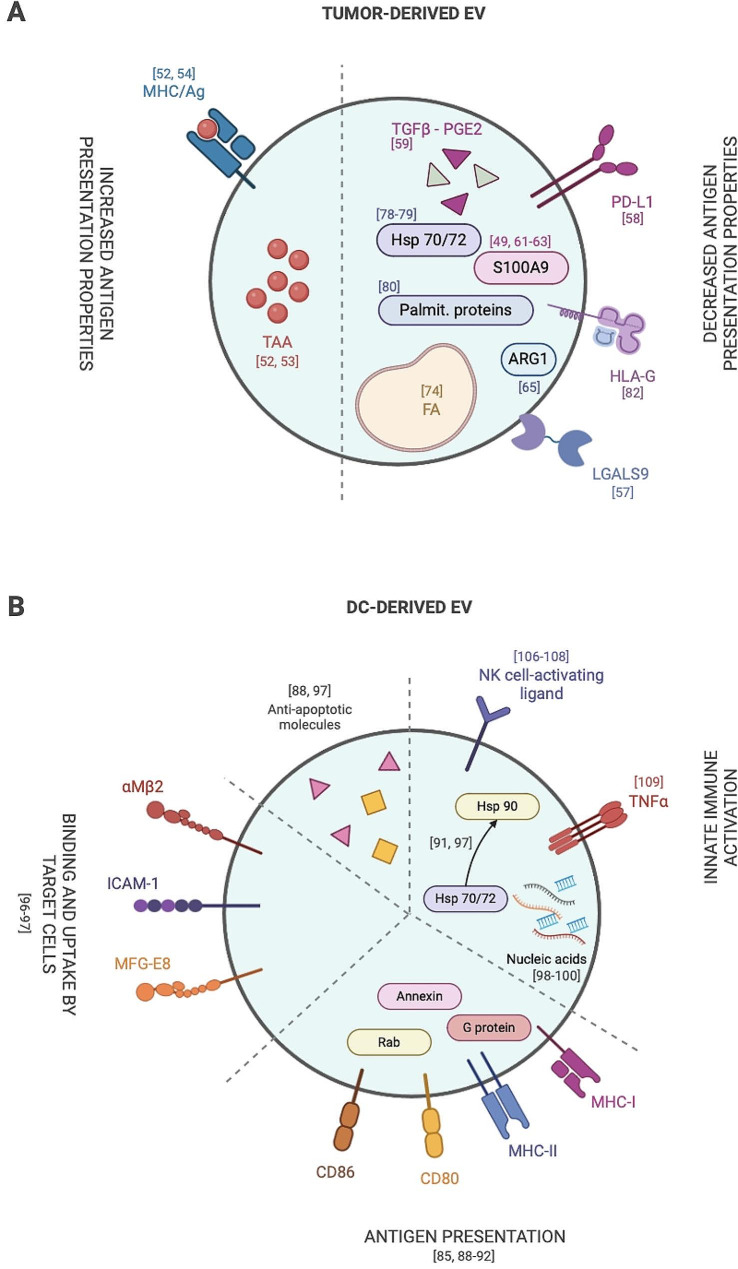
Composition and functions of tumor- and dendritic cell-derived extracellular vesicles. (A)Tumor-derived EVs have been shown two contradictory functional interactions with dendritic cells (DCs): stimulation (depicted on the left) and inhibition of tumor antigen presentation (right side). (B) DC-derived EVs express anti-apoptotic molecules, RNA species that favor innate immune cell activation, molecules involved in antigen presentation and adhesion-associated proteins that enhance EV uptake. Numbers in brackets refer to relevant literature references

Analysis of the literature shows two main and apparently contradictory functional interactions between TEVs and DCs: stimulation and inhibition of the capability to present tumor antigens (Fig. 2A; Table 1).TEVs were originally characterized for their capability to transfer tumor antigens to DCs, thus facilitating antigen presentation and eliciting antigen-specific antitumoral responses [52, 53]. In addition, TEVs were also shown to transfer major histocompatibility complex class I (MHC-I) molecules displaying tumor antigens. This process, known as cross-dressing, has been described for the receptor 2 of human epidermal growth factor (Her2/Neu), the Melanoma Antigen Recognized by T-cells (Mart1), the tyrosinase-related protein (TRP) and the gp100 [52, 54]. In this view, TEVs appeared as promising carriers to deliver antigens in the context of anticancer DC-based vaccination [55]. Indeed, DCs pulsed with TEVs performed better as compared to DCs pulsed with tumor lysates [56].

**Table 1:** Summary of tumor-derived-EV biomarkers found to impact on tumor progression

Origin	Biomarkers	Proposed Mechanisms	Refs
Peritoneal metastasis from melanoma	MHC-I/Mart1 complex	Transfer of tumor antigens to DC to activate T cytotoxic lymphocytes	52
Primary culture of human malignant glioma	MHC-I(HSP70)/MAGE-1 complex	Transfer of tumor antigens to DC to activate T cytotoxic lymphocytes	53
Breast cancer mouse models	EV-loaded with Let-7 family miRNA	TLR7/8 mediated DC or macrophage activation. Promotion of cytotoxic T cell activation	72–73
Chronic lymphocytic leukemia	S-100-A9	Leukemia progression through autocrine NF-kB pathway activation	49
Lung carcinoma and breast cancer cell lines	PD-L1	Inhibition of DC maturation and Th1 activation, Treg induction.	58
Prostate cancer cell line	PGE2	ATP-dependent inhibition of TNFα and IL-12 production by DC. Inhibition of DC-mediated cytotoxic T cell activation	59
Lymph from Cutaneous Melanoma	S-100-A9	Promotion of a pre-metastatic niche in sentinel lymph node by inhibiting DC maturation	61–62
Ascites and plasma of Ovarian Carcinoma	Arginase-1	Metabolic inhibition of CD4 + and CD8 + T-cell proliferation	64
Squamous cell carcinoma cell lines	microRNA	Down-regulation of microRNA mRNA targets to inhibit DC maturation	70
Lung cancer cell lines	microRNA	TLR8-mediated NF-κB activation and secretion of prometastatic inflammatory cytokines.	71
Breast cancer and melanoma cell lines	Long chain fatty acids	Metabolic inhibition of the DC ability to cross-present antigen and activate T cell	74
Plasma of advanced melanoma		Induction of Myeloid Cells showing TGFβ–mediated suppressive activity on T lymphocytes	75
Murine mammary adenocarcinoma cells	TGFbeta and PGE2	Induction of myeloid-derived suppressor cells	76–77
Mouse thymoma and human mammary carcinoma cell lines	HSP70 and 72	Induction of myeloid-derived suppressor cells by autocrine IL-6 production and STAT3 activation	78–79
Acute myeloid leukemia cell lines	Palmitoylated proteins	TLR2-mediated, Akt/mTOR-dependent induction of MDSC	80
Primary cultures of renal cancer stem cells	HLA-G	Inhibition of DC differentiation and maturation	82

More recently, however, the identification of DC-inhibiting molecules expressed by TEVs, together with the general suppression of DC functions often observed in high-grade tumors, suggested that EVs may contribute to immune hijacking rather than immune activation.

One study isolated and fractionated the EV populations (large, medium and small) present in the cerebrospinal fluid of glioblastoma multiforme patients to perform structural and functional comparisons among them and with those of low-grade glioma patients [57]. Of interest, medium and small EVs, but not large EVs, could be efficiently taken up by DCs. In addition, small EVs from glioblastoma, but not medium EVs of either glioblastoma or glioma, nor small EVs from glioma, reduced antigen presentation by DCs, regardless of no observed differences in the content of tumor-associated antigens. Quantitative tandem mass spectrometry analysis of EV cargo revealed that proteome of each fraction was more similar by the type of vesicle rather than by the tumor type, indicating that particle size determines the heterogeneity of EVs in cerebrospinal fluid. Among protein upregulated in glioblastoma small EVs, authors found Galectin 9 (LGALS9), the ligand of the DC-expressed immune checkpoint T-cell immunoglobulin and mucin domain 3 (TIM3). Experiment in LGALS9 knockout mice showed sustained tumor antigen-presenting activity and long-lasting immunity against glioblastoma. To our knowledge, this is the only work directly comparing different EV fractions from different tumors. Most of the literature reviewed in the following paragraphs refers to exosomes or small EVs, despite relevant differences in the techniques used for isolation and characterization, especially in less recent works.

Small EVs produced by lung carcinoma and breast cancer cell lines expressed programmed death-ligand 1 (PD-L1) which, by engaging programmed cell death protein 1 (PD-1) on DCs, decreased their phenotypical maturation and migration to lymph nodes [58]. Prostate cancer-derived EVs induced the release of prostaglandin E_2_ (PGE2) by DCs, which autocrinally upregulated the expression of CD73. This, in turn, induced adenosine triphosphate (ATP)-dependent inhibition of interleukin (IL)-12 and tumor necrosis factor (TNF)-a production and finally also DC-mediated cytotoxic antitumor responses [59]. Also melanoma-derived EVs were shown to reduce DC functions in vitro [60], as recently confirmed in humans by a pioneering study, where EVs where recovered from afferent lymphatic fluid draining from skin melanoma to regional lymph node [61, 62]. EVs were isolated using membrane-affinity spin columns for low sample volumes (< 100 µL) and co-cultured with autologous peripheral blood mononuclear cells (PBMCs) to measure DC expression of cell surface maturation markers CD83 and CD86 by flow cytometry. Interestingly, DC-associated CD83 and CD86 expression was significantly compromised when co-cultured with 2 × 10^10^ but not with 1 × 10^10^ or 1 × 10^9^ lymphatic EVs. The authors identified S100 calcium-binding protein A9 (S100A9) as the responsible for inhibition of DC maturation. Since S100A9 is best known as a damage-associated molecular pattern (DAMP) capable of inducing NF-kB activation by binding to toll-like receptor (TLR) 4 and Receptor for Advanced Glycation Endproducts (RAGE) [63], further studies are awaited to shed light on the molecular mechanisms responsible for the observed block in DC maturation downstream NF-kB activation [61, 63]. In a different tumor type, chronic lymphocytic leukemia, a preponderant role in disease progression was proposed for S100A9 as an activator of the NF-kB pathway. Indeed, when comparing proteomics of plasma EVs from indolent versus progressive leukemia, as well as from individual patients at the onset of disease and during its progression, different protein cargo were found exclusively in patients with progressive leukemias after disease progression. These alterations comprised different networks specific for leukemia progression related to inflammation and oxidative stress, such as NF-kB activation [49]. EVs produced by ovarian carcinoma suppressed T cell proliferation in vitro and in a mouse model, where EVs were described to be transported to draining lymph nodes, taken up by DCs and found to inhibit antigen-specific T-cell proliferation [64]. These EVs contained arginase-1 which, by ʟ-arginine depletion, reduced T-cells expansion as well as antigen presentation by DCs [65]. Interestingly, arginase 1-associated EVs, but not the soluble protein, could inhibit specific T-cell proliferation, highlighting a possible protection of this enzyme from degradation by the EV membrane [64].

EVs are also very well known for their microRNA cargo, which can be delivered to neighboring cells to exert post-transcriptional gene regulation as well as innate immune activation via endosomal TLR stimulation [66–68]. Several tumor-derived microRNAs were described to negatively impact on DC immune functions [69], but their presence in EVs, their actual uptake by DCs and their neat contribution to immunomodulation remain generally poorly known. For example, EVs derived from oropharyngeal squamous carcinoma cell lines were uptaken by DCs and inhibited their differentiation and immune functions, which correlated with the reduction of predicted target genes, but authors could not exclude the contribution of other EV components [70]. Of note, EV-associated TLR-binding microRNAs were originally demonstrated to induce a pro-metastatic inflammatory activation of macrophages that could be reduced in vivo by administrating specific anti-microRNAs [71]. However, tumor EVs loaded with TLR-binding microRNAs, such as the Let-7 family members, could activate DCs and induce an antitumor immune response [72, 73]. Altogether, the EV content and role of microRNAs need further elucidation in each specific cancer context.

Recently, evidence emerged that TEVs may exert metabolic reprogramming of DCs. EV-delivered fatty acids were implicated in DC dysfunction by inducing the expression of peroxisome proliferator-activated receptor, the increase of fatty acids biogenesis and oxidation [74], and finally mitochondrial oxidative phosphorylation and impaired DC functions.

In addition to the suppression of DC immune functions, tumor-derived EVs also induce DC differentiation towards myeloid-derived suppressor cells (MDSCs). In 2006, a first report appeared showing that EVs (at that time, indicated as microvesicles, but corresponding to small EVs based on the separation protocol used) isolated from melanoma cell lines or plasma of patients with advanced melanoma impaired the differentiation of human moDCs in vitro, inducing a phenotype corresponding to MDSCs [75]. A similar result was obtained with murine DCs and mammary adenocarcinoma-derived EVs [76] in vitro and in an in vivo model, where tumor growth was enhanced [77]. In both works, authors demonstrate a role for activated signal transducers and activators of transcription 3 (STAT-3) signaling, already known to inhibit DC differentiation from CD34 + bone marrow progenitors. IL-6 released from MDSCs stimulated by PGE2 and transforming growth factor-β (TGF-β) contained in EVs was responsible for autocrine STAT-3 activation. Other works described that EVs could trigger STAT-3 and MDSCs development and activation because of heat shock proteins (Hsp) 70 and Hsp72, via a TLR2/MyD88-dependent mechanism activating the autocrine production of IL-6 [78, 79]. A general significance of the earlier study was sustained by the use of TEVs obtained from in vitro cultured cell lines of murine mammary adenocarcinoma, thymoma and colon carcinoma or human lung adenocarcinoma, as well as by both in vitro and in vivo experiments on wild type and TLR/MyD88 knockout mice. In addition, MDSCs obtained from cancer patients, treated with a drug inhibiting exosome formation, exhibited reduced suppressor functions. A similar experimental approach was used by other authors to demonstrate that acute myeloid leukemia EVs induce MDSC differentiation in vitro via TLR2 triggering by palmitoylated surface proteins [80]. The involvement of TLR2 stimulation as well as the presence of PGE2 in EVs derived from tumor cell lines were debated between the two groups of authors [51], further underlying the challenges of EV research. Finally, CD105^+^ cancer stem cell-derived EVs (again, a mixture of small and large EVs) were described to express human leukocyte antigen-G (HLA-G), a non-classical MHC-I molecule known to engage inhibitory receptors expressed on T cells, Natural Killer (NK) cells and DCs [81], which retained its ability to inhibit human DC differentiation [82].

In summary, works describing mechanisms of negative DC regulation by EVs clearly overtake in number those describing stimulation (Fig. 2A; Table 1), which is in line with the general suppression of DC functions in tumors. The observed stimulatory properties may depend on the selected target DC population, development stage or subset. Literature analysis also shows contrasting results based on the different tumor derivation or EV preparation [51, 83]. Of particular interest is the hypothesis that studies performed with EVs recovered from cancer patients’ body fluid may be biased by the heterogenous origin of EVs, including EVs released by immune cells and non-cancerous tissues, which may account, at least in part, for the immunostimulatory activity of some preparations [84]. One study compared directly the antigen transferring versus immunosuppressive properties of one EV preparation from prostate cancer cells, confirming immunosuppression as the dominant effect [59].

## Dc-derived EVs: composition and functions

Human DC-derived small EVs can be easily obtained using moDCs differentiated in vitro from CD14^+^ circulating precursors [85, 86]. They are characterized by high levels of sphingomyelin and phosphatidylinositol, which ensure stability in the circulation [87], as well as by classical microdomain-organizing tetraspanin proteins, including CD9, CD37, CD63, CD81, and CD82 [88, 89].

As depicted in Fig. 2B; Table 2, DC-derived small EVs were also reported to expose DC-originating molecules involved in antigen processing and presentation including MHC-I and MHC-II and co-stimulatory molecules such as CD80 and CD86 both in humans and in mice [85, 88–92], which can induce efficient T cell activation ( [93, 94], see further). Not surprisingly, the equipment of DC-derived EVs depends on the activation status of the donor DCs and EVs released from mature DCs were found to display increased levels of intercellular adhesion molecule 1 (ICAM-1), MHC-I and CD86 molecules [95, 96]. In addition, DC-derived EVs were shown to express proteins involved in cross-presentation of lipid antigens such as CD1a, b,c and d [86] and adhesion molecules such as integrin α and β chains (αMβ2), ICAM-1, and milk fat globule EGF factor 8 (MFG-E8), which probably “address” them to target and dock acceptor cells [97]. MFG-E8, in particular, could enhance EV uptake by other antigen presenting cells via the interaction with integrins αvβ3 and αvβ5 [91].

**Table 2:** Summary of DC-derived-EV molecules and their functions

DC-derived EV content	Function	Refs
Annexin II; Gi2a	Exosome function and biogenesis	88–92; 103
MFG-E8; MAC1; CD9	Association to target cells	88–92; 103
MHCI; MHCII; CD86	T cell activation	88–92; 103
MFG-E8	Binds to integrins expressed in dendritic cells and macrophages	102–103
Hsp70	Antitumor effects through citotoxic T lymphocytes	91–97
Hsc73	Antitumor effects Exosome function or biogenesis	91–97
ICAM1	Induce stronger T cell response	96–97
TNFa; FasL; TRAIL	Induce caspase activation and apoptosis in tumor cells Activate NK and IFNg production	109
miRNA	Silencing of transcrits encoding immune-stimulatory molecules Repress target mRNAs of acceptor DCs	98–100
NK ligand (BAT3; NKG2D)	Induce NK innate function Induce cytokine production Induce NK cell activation and proliferation	105–109

EVs also transport cytoplasmic proteins from donor DCs including annexins, RAB proteins and Hsp. In particular, the Hsp70 family members, such as heat shock cognate protein 73 (HSC73), together with Hsp90 family members, where proposed to assist antigen loading on MHC molecules, so enhancing the immunogenicity of EVs [91, 97]. Finally, DC-derived EVs were found to carry signal transduction proteins (G proteins and kinases), anti-apoptotic molecules (thioredoxin peroxidase II, Alix, 14–3–3, and galectin-3) [88, 97] and various RNA species such as mRNA and microRNAs, which were shown to transfer information to bystander DCs but may also regulate other cells either via post-transcriptional mechanisms as well as via the activation of inflammation [98–100].

As for what concerning pDCs, their capability to release EVs remains ill defined, while pDC regulation by EVs is ascertained [68, 101].

**Fig. 3:**
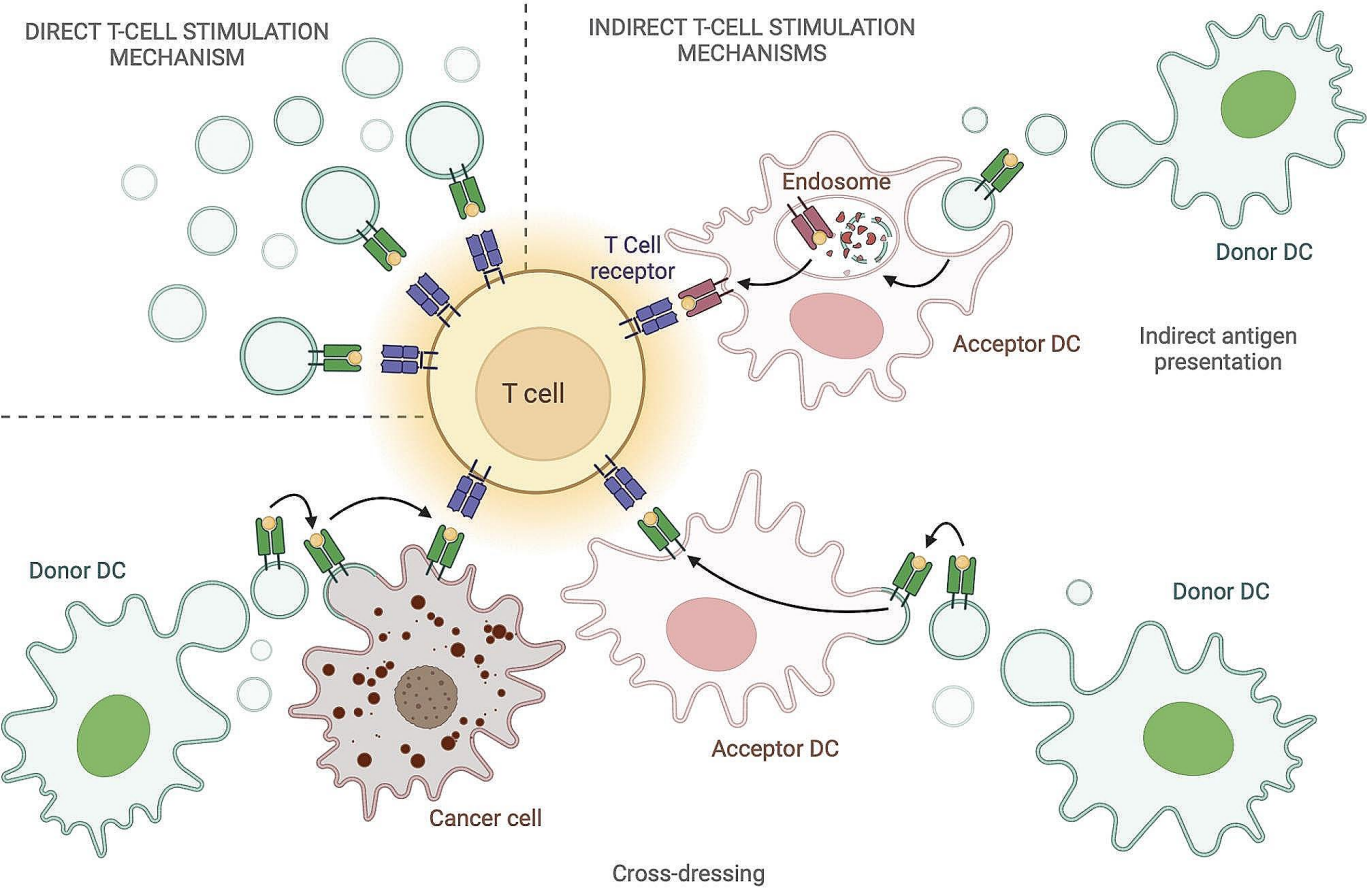
Mechanisms of T cell stimulation by DC-derived extracellular vesicles. DC-derived EVs can present antigens via MHC molecules and stimulate T cell responses either directly or indirectly. Direct T cell stimulation is mediated by peptide/MHC complexes presented on the surface of DC-derived EVs. Indirect T cell stimulation mechanisms include the transfer of antigenic peptide/MHC to bystander DCs or by “cross-dressing”, in which EVs merge to the membrane of the acceptor DCs or cancer cells

DC-derived EVs can present antigens via MHC molecules and stimulate T cell responses either directly or indirectly (Fig. 3). Direct T cell stimulation could be observed in vitro, despite it appeared less efficient as compared to stimulation by donor DCs [92, 102] and better suited for restimulation of activated T cells rather than for priming of naïve T cell [103]. Authors demonstrated that direct stimulation could be ameliorated after EV immobilization and by increasing EV concentration [92, 102]. However, indirect antigen presentation, meaning the transfer of antigenic peptide/MHC complexes to bystander APCs, seems a more efficient mechanism of T cell activation by DC-derived EVs [103]. Another mechanism of indirect T cell stimulation is the previously described “cross-dressing”, where DC-derived EVs transfer the peptide/MHC complexes to an acceptor APC after merging to its membrane [89, 90, 104]. This, in turn, allows the immediate recognition of MHC-presented peptides by T cells, without the need for antigen processing [103]. Finally, DC-derived EVs can exert indirect T cell stimulation by transferring MHC-peptide complexes to tumor cells, which are then targeted directly by T cells [105]. A recent work demonstrated that pDCs can cross-prime naïve CD8^+^ T cells by transferring antigens to bystander cDCs via the release of EVs [106].

Increasing evidence is now showing that DC-derived EVs can also trigger innate immune response by expressing NK cell-activating ligands, which can engage natural killer group 2D (NKG2D) and natural killer protein 30 (NKp30) on NK cells and activate their cytotoxic functions, and also by increasing the number of circulating NK cells when administered therapeutically [105, 107]. In a melanoma mouse model, DC-derived EVs were shown to induce NK cell proliferation and activation through both NKG2D and IL15Ralpha engagement [108]. Finally, DC-derived EVs express transmembrane TNF-α, which can directly activate NK cells and stimulate them to secrete IFN-γ, a potent immunoactivatory cytokine [109].

## Diagnostic and prognostic value of EVs in cancer

The retrieval of EVs in the liquid compartments of the human body underlies their inclusion as integral components of the so-called liquid biopsy. In this view, the analysis of tumor associated EV cargo found in blood, saliva or urine has been exploited to expand the panel of diagnostic biomarkers used in oncology. In fact, EVs satisfy many of the characteristics of an ideal biomarker such as the ability to distinguish cancers from benign diseases and healthy conditions. Several studies have pointed out that EV cargo from cancer cells is significantly different respect to the one from healthy cells. Reported differences encompass a wide range of molecules including proteins such as transcription factors, signalling molecules, enzymes and membrane receptors [110]. Also, lipids, glycan and nucleic acids (miRNA, mRNA and DNA) have been described differentially expressed in cancer cells as recently reviewed [111–113]. Further relevant aspects of the EV components as diagnostic markers reside in their long half-life, that could be due to the ability of the EV membrane to protect the cargo from degradation [64]. In addition, EVs carry molecules that are cell-type specific, allowing to better understand the origin of tumor cells and, finally, being continuously released by cancer cells, EVs are also abundant in the circulation thus increasing the chance to detect low tumour burdens [110, 114]. Being a snapshot of tumor cells, EV cargo may also reflect changes in tumor behaviour and, accordingly, showing a potential prognostic value to estimate metastasis propension, patient survival and tumor recurrency. Several studies provided exosomal protein or miRNA signatures to identify cancer patients at risk for metastasis at both specific or non-specific distant sites. Tumour-derived exosomes were shown to reshape distant microenvironments driving organ-specific metastasis or promoting pre-metastatic niches. Organotropic integrins expressed by tumour exosomes were found directly involved in the determination of the preferred site of metastasis from different cancer cells [115]. Moreover, in patients with lung and breast cancer, exosomes secreted by brain metastatic tissues and carrying the CEMIP protein, promoted the development of a pro-metastatic environment, leading to accelerated metastasis growth and reduced patient survival [116]. Similarly, pancreatic cancer-derived exosomes were found involved in the formation of a pre-metastatic niche promoting liver metastasis [117, 118]. Melanoma-derived exosomes also induced vascular leakiness at pre-metastatic sites and educated bone marrow progenitors of DCs toward a pro-vasculogenic and pro-metastatic phenotype [119]. A microarray analysis of miRNA cargo in serum exosomes from colorectal cancer patients identified a miRNA signature correlated with poor prognosis and recurrence [120]. Overall, literature data have demonstrated that the knowledge of EV components could play a relevant role in Oncology, especially in the light of novel personalized approaches.

## Applications of EVs in cancer immunotherapy

Despite often loaded with DC-suppressing molecules, TEVs still represent a privileged system to deliver antigenic material to DCs in the context of immunotherapy and DC-based vaccine design. Interestingly, the anti-tumor activity of EV-pulsed DCs appears to be much greater as compared to that of DCs loaded with tumor lysates [56]. To maximize the benefits of increased antigen presentation, EVs can be engineered to “customize” cargoes and block the influence of negative DC-regulators. In other words, engineered exosomes lacking inhibitory molecules are expected to perform better as inducers of antitumor DCs. For example, DCs loaded with EV depleted in TGF-β, or enriched in IL-12, supported stronger induction of anti-tumor immune responses as compared to unmodified TEVs [121, 122]. Other studies show DC-stimulating properties when EVs are loaded with TLR-activating microRNAs, such as the Let-7 family members [72, 73]. Needless to say, the clinical relevance of this approach strictly depends on the precise knowledge of specific inhibitory mechanisms and of the balance between induction and inhibition exerted by EVs in each tumor type and condition.

As compared to TEVs, DC-derived EVs seem more promising tools for anticancer vaccines which also possess several advantages as compared to DC-based drugs [123]. First, they contain 10–100 folds more tumor-associated antigens and MHC molecules in comparison to DCs and are less sensitive to immunosuppressive mediators of the TME. In addition, EVs are smaller and less complex than entire cells, which renders them easier to manipulate in order to modify the cargo or the tumor-addressing molecules. Finally, EVs have a longer life span and shelf life as compared to whole cells [103].

Two Phase I clinical trials were conducted with MHC I/II loaded DC-derived EVs, in advanced melanoma and non-small cell lung carcinoma (NSCLC) patients [105, 124]. In the first study, thirteen HLA-A2 positive NSCLC patients with high expression of melanoma-associated antigen encoding (MAGE)-A3/A4 antigens were treated with an acceptable safety profile. In two patients a stable disease was observed and was sustained for over 12 months. The second Phase I trials included fifteen MAGE3 positive metastatic melanoma patients and the overall results included two stable diseases, one minor, one partial and one mixed response. Of note, in both the studies no Delayed-Type Hypersensitivity (DTH) nor MAGE-specific T cell response could be observed, and clinical responses were attributed to increased NK cell effector functions [105, 108]. However, the absence of T cell-specific anti-tumor immune responses needs to be taken into consideration as a major explanation of the observed limited clinical efficacy, despite other possible causes such as the advanced cancer stage, the limited number of recruited patients, who also received previous therapies, and the inappropriate preselection criteria [105].

To improve the therapeutic efficacy and specificity of DC-derived EVs, their combined use along with immune checkpoint inhibitors, namely anti- Cytotoxic T-lymphocyte associated protein4 (CTLA4), anti-PD-1 or anti-PD-L1, was recently proposed and proved to represent a valuable therapeutic approach [125, 126]. Exosomes prepared from Ovalbumin-pulsed, activated DCs and modified with anti-CTLA4 antibody to block this inhibitory molecule resulted very efficient in boosting the cytotoxic T cells/T reg ratio within the tumor and increase the production of IFN-γ and TNF-α in both sera and tumors as a result of increased T cell activation and infiltration into the tumor [125].

Another strategy to improve anti-tumor immune responses by DC-derived EVs, based on previous observations of increased immunostimulatory properties by IFN-γ DC-derived exosomes [95], was elaborated in a Phase II clinical trial where EVs were derived from IFN-γ stimulated DC loaded with MHC class I- and II-restricted cancer antigens [127]. Twenty-two advanced HLA2 + NSCLC patients were treated after metronomic low-dose chemotherapy aimed at reducing T regulatory cells and inducing IFN-g/IL-17 producing T cells. Despite seven patients exhibited stable disease, the primary endpoint of at least 50% of patients with progression-free survival at 4 months after the end of chemotherapy could not be met. Notably, an improvement of NKp30-dependent NK cell functions was observed after IFN-γ DC-derived exosome injections in patients experiencing a longer progression free survival (PFS) [127].

Finally, a pDC cell line-cancer vaccine, not derived from autologous pDCs, was recently described and demonstrated to induce an efficient T cell-mediated anti-tumor response in a clinical trial on melanoma patients [128]. However, future studies are needed to better characterize small EVs derived from immortalized pDC cell lines and to explore their large-scale production and their potential application as cancer vaccines.

## Conclusions and future perspectives

The role of EVs stands high in the future of cancer immunotherapy either as targets (especially TEVs) and therapeutic tools (especially DC-derived EVs). As tools, EVs are biogenic nanocarriers with good biocompatibility, biodegradability and safety, which therapeutic application mimics a nature’s delivery system. However, the clinical application of EVs is still hampered by several problems. The first, and maybe major challenge is posed by our incomplete understanding of EV biology and cargoes, as discussed in the first part of this review: indeed, EVs have multiple mechanisms of action, targets and effects, with results on the immune response that are not yet fully understood. Second, we need to determine the pharmacokinetic of the injected therapeutic EVs to make sure that they reach T cell zones of secondary lymphoid organs in acceptable quantities [129]. Third, standardized methods for high-throughput isolation and purification of “physiologic” EVs are still missing. For what concerning TEVs, these are currently separated from the supernatant of incubated cells with concerns regarding yield and purity [130], while DC-derived EVs are usually prepared from autologous moDCs, which can be derived in vitro in relatively larger numbers as compared to primary DCs but may display different migratory and T-cell activating capabilities leading to unsatisfactory clinical effects [131]. One interesting approach might be the establishment of immortalized cell lines, based on the positive results of clinical trials achieved by pDC cell line-based vaccines [128]. In general, the lack of standardized guidelines of production and application represents a tremendous challenge for the future of EV-based therapy and the formulation of guidelines of good manufacturing practice remains an urgent need.

Despite many obstacles, especially DC-derived EVs remain highly promising candidates in cancer immunotherapy that may replace cell-based strategies in the fullness of their time.

## Data Availability

Not available.

## Abbreviations

APC: Antigen-Presenting Cells
ATP: Adenosine Triphosphate
CD: Cluster of Differentiation
cDCs: conventional Dendritic Cells
CTLA4: Cytotoxic T-lymphocyte Associated Protein 4
DAMP: Damage-Associated Molecular Pattern
DCs: Dendritic Cells
DTH: Delayed-Type Hypersensitivity
EVs: Extracellular Vesicles
Her2: Human Epidermal Growth Factor Receptor 2
HLA: Human Leukocyte Antigen
HSC73: Heat Shock Cognate Protein 73
Hsp: Heat Shock Protein
ICAM-1: Intercellular Adhesion Molecule 1
IFNs: Interferons
IL: Interleukin
ILVs: Intraluminal Vesicles
LGALS9: Galectin 9
MAGE: Melanoma-Associated Antigen Encoding
Mart1: Melanoma Antigen Recognized by T-cells 1
MDSCs: Myeloid-Derived Suppressor Cells
MFG-E8: Milk Fat Globule EGF Factor 8
MHC: Major Histocompatibility Complex
moDCs: monocyte-Derived Dendritic Cells
mRNA: messenger RNA
MVBs: Multivesicular Bodies
MyD88: Myeloid Differentiation Primary Response Protein 88
NF-kB: Nuclear Factor Kappa-Light-Chain-Enhancer Of Activated B cells
NK: Natural killer
NKGD2: Natural Killer Group 2D
NKp30: Natural Killer Protein 30
NSCLC: Non-Small Cell Lung Carcinoma
PBMCs: Peripheral Blood Mononuclear Cells
pDCs: plasmacytoid Dendritic Cells
PD-1: Programmed Cell Death Protein 1
PD-L1: Programmed Death-Ligand 1
PFS: Progression Free Survival
PGE2: Prostaglandin E_2_
PRRs: Pattern Recognition Receptors
RAGE: Receptor for Advanced Glycation Endproducts
S100A9: S100 calcium-binding protein A9
STAT3: Signal Transducers And Activators Of Transcription 3
TEVs: Tumor-Derived Extracellular Vesicles
TGF: Transforming Growth Factor
TIM3: T-cell Immunoglobulin And Mucin Domain 3
TLR: Toll-Like Receptor
TME: Tumor Micro-Environment
TNF: Tumor Necrosis Factor
TRP: Tyrosinase-Related Protein
